# Nitrogen source-dependent inhibition of yeast growth by glycine and its *N*-methylated derivatives

**DOI:** 10.1007/s10482-019-01342-z

**Published:** 2019-10-19

**Authors:** Tomas Linder

**Affiliations:** Department of Molecular Sciences, Swedish University of Agricultural Sciences, Box 7015, 750 07 Uppsala, Sweden

**Keywords:** Adaptive response, Antifungal, Metabolism, Phenotype, Yeast

## Abstract

The effect of nitrogen source on the inhibitory properties of glycine and its *N*-methylated derivatives *N*-methylglycine (sarcosine), *N*,*N*-dimethylglycine, *N*,*N*,*N*-trimethylglycine (glycine betaine) on yeast growth was investigated. On solid minimal medium, all four glycine species completely or partially inhibited growth of *Kluyveromyces lactis*, *Komagataella pastoris*, *Ogataea arabinofermentans*, *Spathaspora passalidarum* and *Yamadazyma tenuis* at concentrations 5–10 mM when 10 mM NH_4_Cl was the sole source of nitrogen. If NH_4_Cl was substituted by sodium L-glutamate as the sole source of nitrogen, obvious growth inhibition by glycine and its *N*-methylated derivatives was generally not observed in any of these species. No obvious growth inhibition by any of the glycine species at a concentration of 10 mM was observed in *Cyberlindnera jadinii*, *Lipomyces starkeyi*, *Lodderomyces elongisporus*, *Scheffersomyces stipitis* or *Yarrowia lipolytica* on solid minimal medium irrespective of whether the nitrogen source was NH_4_Cl or sodium L-glutamate. Growth inhibition assays of *K. pastoris* in liquid minimal medium supplemented with increasing concentrations of *N,N*-dimethylglycine demonstrated inhibitory effects for nine tested nitrogen sources. In most cases, *N,N*-dimethylglycine supplementation caused a decrease in growth efficiency that appeared to be proportional to the concentration of *N,N*-dimethylglycine. The biological relevance of these results is discussed.

## Introduction

Glycine is the simplest proteinogenic α-amino acid as well as a central intermediate in cellular one-carbon metabolism (Wang et al. 2013). In several domains of life, glycine is also metabolically linked to the quarternary amine choline through a series of three methylation/demethylation reactions as well as two oxidation/reduction reactions. The glycine-choline metabolic axis serves different purposes in different domains of life and is sometimes incomplete. Of the four intermediates within the glycine-choline metabolic axis, *N,N,N*-trimethylglycine (glycine betaine) is itself a common end product as a compatible solute in archaea, bacteria and many eukaryotic organisms (Chen and Murata 2008; Cleland et al. 2004).

The utilisation of glycine as a nitrogen source among budding yeasts (phylum Ascomycota, sub-phylum *Saccharomycotina*) has been demonstrated in the common baker’s yeast *Saccharomyces cerevisiae* and involves the glycine cleavage complex (McNeil et al. 1997). Notably not all species of budding yeast can utilise glycine as a nitrogen source (Linder 2014). *N*-methylated species of glycine can be utilised as nitrogen sources by some species of budding yeasts (Linder 2014; Middelhoven et al. 1991) but the exact pathway and enzymes involved remain to be identified. Budding yeasts appear to synthesise the compatible solute glycerophosphocholine rather than glycine betaine in response to environmental stress (Kiewietdejonge et al. 2006) although the presence of glycine betaine in yeast extract has been reported (Dulaney et al. 1968).

A previous study by this author had observed an inhibitory effect of glycine and its *N*-methylated derivatives on growth in some species of yeast under conditions of nitrogen limitation (Linder 2014). *N,N*-dimethylglycine has also been previously identified in a screen for growth inhibitors of *S. cerevisiae* (Kolaczkowski et al. 1998) although this initial observation has not been further characterised. Glycine has been shown to inhibit bacterial growth at higher concentrations (Gordon and Gordon 1943; Hishinuma et al. 1969; Minami et al. 2004). In bacteria, the inhibitory mechanism appears to involve interference with synthesis of the peptidoglycan cell wall (Hammes et al. 1973). However, yeast cell walls differ fundamentally from that of bacteria both in terms of structure and composition with the main structural components being β-glucan and chitin (Latgé 2007). The aim of the present study was therefore to further characterise the inhibitory effects of glycine and its *N*-methylated derivatives on yeast growth and investigate how nitrogen availability affects the degree of growth inhibition.

## Materials and methods

### Yeast strains and reagents

The yeast strains *Cyberlindnera jadinii* CBS 5609, *Kluyveromyces lactis* CBS 2359, *Komagataella pastoris* CBS 704, *Lipomyces starkeyi* CBS 1807, *Lodderomyces elongisporus* CBS 2605, *Ogataea arabinofermentans* CBS 8468, *Scheffersomyces stipitis* CBS 6054, *Spathaspora passalidarum* CBS 10155, *Yamadazyma tenuis* CBS 615 and *Yarrowia lipolytica* CBS 7504 were purchased from the Westerdijk Fungal Biodiversity Institute (Utrecht, the Netherlands). γ-amino butyric acid (GABA), D-alanine, L-alanine, L-proline, potassium L-aspartate, sodium L-glutamate, urea and the hydrochloride salts of ethylamine, glycine, sarcosine, *N*,*N*-dimethylglycine and glycine betaine were purchased from Sigma Aldrich. All compounds were dissolved in water at high concentration (0.4–2 M), sterilised through filtration and stored as 1-ml aliquots at – 20 °C.

### Yeast growth assays

All growth inhibition assays employed a reduced sulfur/nitrogen-limited glucose medium (RSNLD), which only contains trace amounts of nitrogen in the form of vitamins (Linder 2018). Solid media growth assays used agarose (20 g l^–1^) instead of agar as a gelling agent in order to limit the introduction of cryptic nitrogen sources. For growth assays on solid medium, yeast strains were pre-cultured overnight in 3 ml minimal glucose medium (MMD), which consists of 6.7 g Difco yeast nitrogen base without amino acids (Becton, Dickinson and Company) l^–1^ and 20 g glucose l^–1^. Pre-cultured cells were then washed once in sterile water and subsequently diluted to OD_600_ 0.1 in sterile water. 2 μl of this cell suspension was spotted onto RSNLD agarose plates containing the indicated concentrations of primary nitrogen source and either glycine, sarcosine, *N*,*N*-dimethylglycine or glycine betaine. Plates were incubated at 30 °C and photographed after 6 days. *K. pastoris* CBS 704 growth assays in RSNLD liquid media were essentially performed as described previously (Linder 2018) with a total cultivation volume of either 3 or 6 ml as indicated in the text. Each nitrogen source was present at a final concentration of 10 mM total nitrogen. *N,N*-dimethylglycine was included as indicated from a sterile 1 M stock solution in water. Each growth assay was performed in triplicate with each biological replicate derived from a separate pre-culture.

## Results

Previous work by the author had demonstrated that glycine and its *N*-methylated derivatives could inhibit growth of some species of yeast under conditions of nitrogen limitation (Linder 2014). The previous study had employed a chemically defined minimal medium lacking a conventional nitrogen source with only trace amounts of nitrogen in the form of the vitamins 4-aminobenzoate, biotin, nicotinate, pantothenate, pyridoxine, riboflavin and thiamine. It has yet to be established which of these vitamins can be utilised as nitrogen sources by yeasts but the majority of yeast species would display very weak but detectable growth in the range of 0.03–0.1 OD_600_ units over an 18-day period. However, if 10 mM glycine or the equivalent concentration of any of its three *N*-methylated derivatives was added to the medium, those yeast species who were unable to utilise the particular glycine variant as a nitrogen source would generally fail to show any detectable growth (Linder 2014).

As nitrogen had been limited in the previous study, the first aspect to be investigated in the present study was whether an inhibitory effect would also be observed when nitrogen was not limiting. A simple semi-quantitative spot growth assay on solid medium was therefore employed to investigate potential inhibitory effects by glycine and its *N*-methylated derivatives under nitrogen non-limiting conditions. It had been observed that agar appeared to contain cryptic sources of nitrogen that could support marginal growth on solid minimal medium that had not been supplemented with an external nitrogen source (author’s unpublished observation). Therefore agarose rather than agar was used as a gelling agent in assays involving solid media. L-glutamate and NH_4_Cl were selected as nitrogen sources to be tested at this stage with non-supplemented medium used as an experimental control. Ten species of budding yeast were selected for further characterisation of potential inhibitory effects by glycine and its *N*-methylated derivatives. All ten species had been included in the previous study by the author, which had demonstrated growth inhibition during nitrogen limitation (Linder 2014). The selected species spanned the taxonomic range of budding yeasts and were therefore expected to provide a representative cross-section of lineage-specific responses to glycine and its *N*-methylated derivatives.

**Fig. 1:**
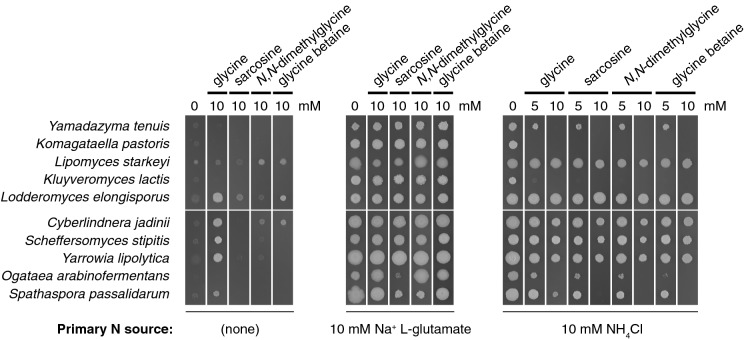
Growth inhibition of different yeast species by glycine and its *N*-methylated derivatives on solid media. Agarose (20 g l^–1^) was used instead of agar to exclude traces of cryptic nitrogen sources. Strains were pre-cultured overnight in 3 ml MMD broth and then diluted to OD_600_ 0.1 in sterile water. 2 μl cell suspension of each strain was spotted on solid RSNLD medium with the indicated concentration of primary nitrogen source and glycine or one of its *N*-methylated variants. Plates were incubated for 6 days at 30 °C and then photographed

The results of the spot growth assay demonstrated both species-specific and nitrogen source-specific differences in the effects on growth by glycine and its *N*-methylated derivatives (Fig. 1). The growth of *C. jadinii*, *Lip. starkey*i, *Lod. elongisporus*, *Sch. stipitis* and *Yar. lipolytica* appeared largely unaffected by the inclusion of 10 mM glycine or any of its *N*-methylated derivatives irrespective of whether L-glutamate or NH_4_Cl were provided as a nitrogen source. *C. jadinii*, *Lip. starkey*i, *Lod. elongisporus*, *Sch. stipitis*, *Spa. passalidarum* and *Yar. lipolytica* also displayed growth on the non-supplemented control in the presence of 10 mM glycine, which is consistent with the ability of these species to utilise glycine as a nitrogen source (Linder 2014). *Klu. lactis* and *K. pastoris* displayed no detectable growth in the presence of 5 mM glycine or any of its *N*-methylated derivatives when NH_4_Cl was provided as a nitrogen source while no obvious growth inhibition was observed if L-glutamate was provided as a nitrogen source instead. *O. arabinofermentans*, *Spa. passalidarum* and *Yam. tenuis* displayed intermediate sensitivity with weak but detectable growth in the presence of 5 mM glycine or any of its *N*-methylated derivatives when NH_4_Cl was provided as a nitrogen source but no detectable growth at 10 mM.

*K. pastoris* was selected for further study to investigate the effect of nitrogen source on sensitivity to growth inhibition by glycine and its *N*-methylated derivatives. These growth assays were performed in liquid medium to better resolve the inhibitory effect. Unlike growth assays on solid media, measurement of microbial growth in liquid media enables the identification of effects on growth variables such as growth lag, growth rate and growth efficiency (Warringer et al. 2008). Since the magnitude of inhibition by glycine and its *N*-methylated derivatives had appeared roughly equal when assayed on solid medium, only *N,N*-dimethylglycine was included in the liquid growth assays. *N,N*-dimethylglycine was selected in part because of a previous report of inhibitory activity in yeast (Kolaczkowski et al. 1998), although this result had not been characterised further. Seven nitrogen sources in addition to L-glutamate and NH_4_Cl were selected for assaying the effects of *N,N*-dimethylglycine on *K. pastoris* growth in minimal medium. All nine nitrogen sources are assimilated through separate pathways (Linder 2019b). Three concentrations of *N,N*-dimethylglycine – 2, 5 and 10 mM, were tested for each nitrogen source and assays lasted 18 days with growth analysis by OD measurement every 6 days.

**Fig. 2:**
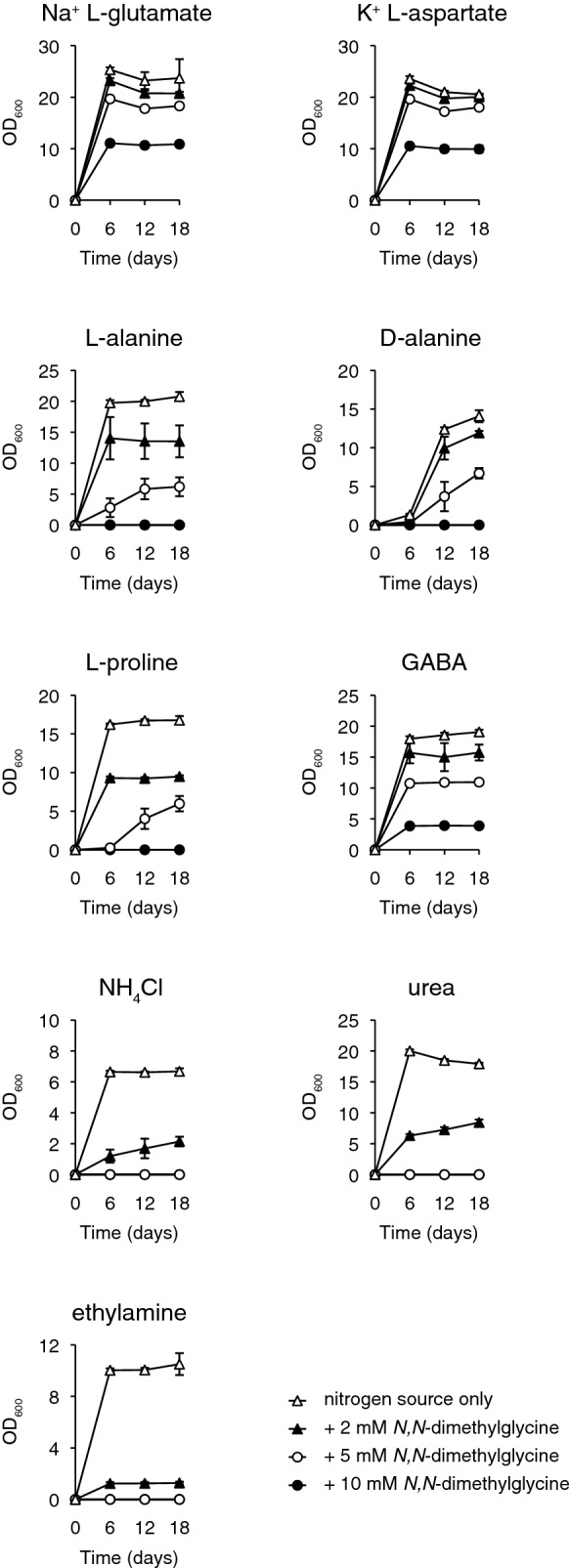
Dose–response curves demonstrating nitrogen source-dependent growth inhibition of *K. pastoris* by *N,N*-dimethylglycine in liquid media. *K. pastoris* was cultured in 3 ml RSNLD medium supplemented with 10 mM total nitrogen of the indicated nitrogen source (initial OD_600_ 0.005) and the indicated concentration of *N,N*-dimethylglycine. Samples were incubated in a shaker set at 30 °C, 200 rpm., and OD_600_ was measured after 6, 12 and 18 days. Growth assays were performed in triplicate with error bars indicating one standard deviation

*K. pastoris* appeared to have reached stationary phase already after 6 days for all tested nitrogen sources (with the exception of D-alanine) in the absence of *N,N*-dimethylglycine supplementation (Fig. 2). The observed differences in final cell density between individual nitrogen sources without *N,N*-dimethylglycine supplementation likely reflects the corresponding differences in energetic costs for assimilation as well as the caloric value of any remaining carbon skeleton following de- or transamination (Linder 2019b). For example, the assimilation of L-glutamate, L-aspartate and GABA results in concurrent production of the citric acid cycle intermediates α-ketoglutarate, oxaloacetate and succinate, respectively, which can all serve as additional carbon sources. In contrast, the assimilation of ammonia by glutamate dehydrogenase or glutamine synthetase requires an input of energy in the form of NADPH or ATP, respectively, without simultaneous production of a potential carbon source (Linder 2019b). This could explain why ammonia-grown *K. pastoris* cells displayed the lowest cell density of all tested nitrogen sources in the current study.

The nine tested nitrogen sources could be divided into three groups based on the resulting magnitude of *N,N*-dimethylglycine inhibition. The first group included *K. pastoris* cultures supplemented with either L-glutamate, L-aspartate or GABA, which all still displayed detectable growth with 10 mM *N,N*-dimethylglycine. The second group included *K. pastoris* cultures supplemented with either L-alanine, D-alanine or L-proline, which all still displayed growth at 5 mM *N,N*-dimethylglycine but not at 10 mM. The third group included *K. pastoris* cultures supplemented with either NH_4_Cl, ethylamine or urea, which all still displayed growth at 2 mM *N,N*-dimethylglycine but not at 5 mM or higher. It was notable that *K. pastoris* cultures supplemented with L-glutamate displayed detectable dose-dependent inhibition to *N,N*-dimethylglycine in liquid medium, which had not been obvious in the solid medium growth assay. This difference was ascribed to the difference in yeast growth dynamics between solid and liquid media and could also be a result of the difference in the size of starting inoculum of the two different assays.

Some degree of inhibition was observed in all assays with an apparent dose-dependent decrease in growth efficiency. One notable exception was cells growing on L-proline as the sole nitrogen source in the presence of 5 mM *N,N*-dimethylglycine, where the effect appeared to affect the growth rate but not the efficiency. In the majority of remaining cases, the difference in final OD_600_ between untreated and treated samples (ΔOD_600_) appeared to be roughly proportional to the *N,N*-dimethylglycine concentration.

As growth had only been assayed after 6 days, there was insufficient data to assess whether there was additional cases of extended lag phase or decreased growth rate. The growth assay was therefore repeated for those concentrations of *N,N*-dimethylglycine where growth could be observed for all nine nitrogen sources. In order to capture the dynamics of the initial stages of growth, cell density was measured every 24 h up to 5 days following inoculation.

**Fig. 3:**
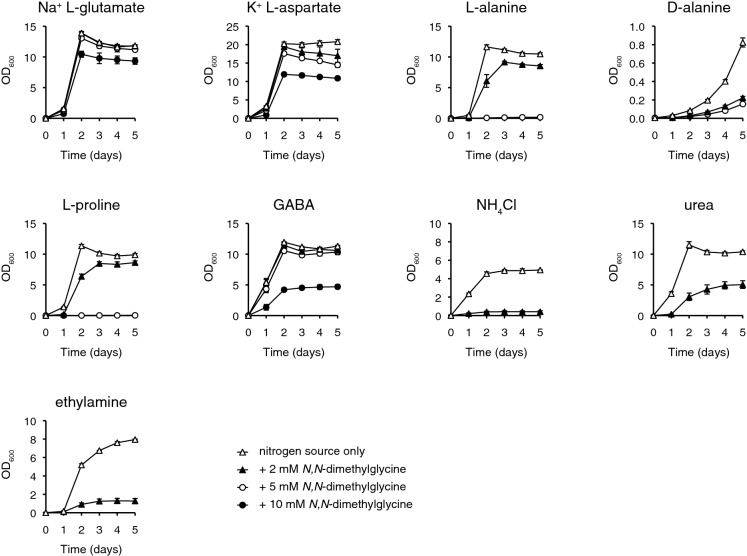
Dose-response curves demonstrating nitrogen source-dependent growth inhibition of *K. pastoris* by *N,N*-dimethylglycine in liquid media. *K. pastoris* was cultured in 6 ml RSNLD medium supplemented with 10 mM total nitrogen of the indicated nitrogen source (initial OD_600_ 0.005) and the indicated concentration of *N,N*-dimethylglycine. Samples were incubated in a shaker set at 30 °C, 200 rpm, and OD_600_ was measured every 24 h. Growth assays were performed in triplicate with error bars indicating one standard deviation

The early-stage growth dynamics of *K. pastoris* in the presence of *N,N*-dimethylglycine appeared to largely agree with the previous assay of later stages of growth (Fig. 3). However, it was possible to resolve some instances of growth lag that had not been detectible in the previous assay, which included urea-grown cells supplemented with 2 mM *N,N*-dimethylglycine, L-proline-grown cells supplemented with 2 mM *N,N*-dimethylglycine and L-alanine-grown cells supplemented with 5 mM* N,N*-dimethylglycine. It was notable that just as the sensitivity to *N,N*-dimethylglycine depended on the nitrogen source, so did the initial growth dynamics. For example, both L-alanine- and L-proline-grown cells displayed an extended growth lag when supplemented with 5 mM* N,N*-dimethylglycine that was not observed in any of the other tested nitrogen sources.

## Discussion

Pathways that catalyze the interconversion of glycine and its *N*-methylated derivatives sarcosine, *N,N*-dimethylglycine and glycine betaine have been well studied in bacteria, animal, plants and filamentous fungi (Cleland et al. 2004; Lambou et al. 2013). However, these pathways appear to be absent in budding yeasts although some yeast species appear to utilise some of these compounds as nitrogen sources (Linder 2014). The well-established role of glycine betaine as a compatible solute in plants, bacteria and archaea has lead to the general perception that this compound is beneficial to living cells. Likewise, glycine is one of twenty proteinogenic α-amino acids and as such would not be expected to be detrimental to cell growth. Previous reports of inhibitory effects by glycine in bacteria were observed under relatively high glycine concentrations (Gordon and Gordon 1943; Hammes et al. 1973; Hishinuma et al. 1969). The author had previously observed inhibitory effects by glycine and its *N*-methylated derivatives under conditions of nitrogen limitation (Linder 2014). In addition, a high-throughput screen of potential cytostatic and cytotoxic compounds in *S. cerevisiae* had identified *N,N*-dimethylglycine as a growth inhibitor on rich medium in a *S. cerevisiae* strain with a defective xenobiotic response (Kolaczkowski et al. 1998). However, the inhibitory effect of *N,N*-dimethylglycine had not been characterised further in this previous study.

The current study demonstrated that glycine and its *N*-methylated derivatives at concentrations as low as 5–10 mM can inhibit growth of several species of yeast on solid chemically defined minimal medium when 10 mM NH_4_Cl was provided as a nitrogen source. The inhibitory effect was largely absent if NH_4_Cl was substituted by L-glutamate although it is possible that some small degree of growth inhibition by glycine and its *N*-methylated derivatives could still occur as suggested by subsequent liquid growth assays of *K. pastoris*. The mechanism of inhibition by glycine and its *N*-methylated derivatives remains to be determined. Sarcosine has previously been shown to compete with L-proline import in the yeasts *Kluyveromyces marxianus* (Magaña-Schwencke et al. 1973) and *Candida albicans* (Dabrowa and Howard 1981). However, this mechanism of inhibition would seem unlikely since inhibition was observed on all nine tested nitrogen sources, which employ separate import systems. In addition, simple competition for transporters would be expected to decrease growth rate but not growth efficiency (Warringer et al. 2008). One would expect some discernable growth even at the highest *N,N*-dimethylglycine concentrations, which are at most equimolar to the nitrogen source. The dose-dependent decrease in growth efficiency observed in *K. pastoris* in the presence of all nine tested nitrogen sources would suggest that the yeast is unable to detoxify the inhibitor by metabolic means and must therefore expend energy to continuously expel the compound from the cell interior using xenobiotic efflux pumps. Such an expenditure of energy reserves would consequently leave less resources available for cell growth, which would result in the observed decrease in final cell density.

Two possible hypotheses were considered that could explain the differences in magnitude of inhibition depending on the nitrogen source. The first hypothesis is that the nitrogen source determines the complement of transporters at the cell surface, which in turn determines the efficiency of *N,N*-dimethylglycine import. The second hypothesis is that the susceptibility to glycine and its *N*-methylated derivatives depends on the metabolic configuration of the cell (what pathways are active and their relative flux), which is also expected to be governed by nitrogen source availability. Identification the underlying inhibitory mechanism of glycine and its *N*-methylated derivatives may shed light on whether either of these hypotheses reflect the true influence of the nitrogen source on the magnitude of growth inhibition. Two genetic approaches could be employed to identify the cellular target or targets of inhibition. The first approach would be genomic re-sequencing of spontaneous mutants displaying increased tolerance to these compounds. The second genetic approach would be to perform a suppressor screen using a genomic plasmid library in a plasmid-compatible system such as *S. cerevisiae*.

It was notable that growth inhibition could be observed in *K. pastoris* already at 2 mM* N,N*-dimethylglycine, which demonstrates the potency of these compounds. Apart from the previous study by the author (Linder 2014) and a preliminary observation in large-scale study using attenuated strains of *S. cerevisiae* (Kolaczkowski et al. 1998), an extensive review of the published literature failed to identify reports of growth inhibition in yeast by glycine or its *N*-methylated derivatives. However, it has previously been shown that pre-incubation of yeast in glycine betaine increases stress tolerance in the biocontrol yeasts *Cystofilobasidium infirmominiatum* (Liu et al. 2011a) and *Candida oleophila* (Sui et al. 2012). Likewise, supplementation with glycine or glycine betaine has been shown to increase substrate utilisation and viability of *S. cerevisiae* in high-gravity fermentations (Thomas et al. 1994), which subject the yeast cells to osmotic stress. All three studies mentioned above had discussed the observed effects of glycine betaine supplementation in the context of its ability to act as a compatible solute that could potentially be acquired by the yeast cell from the external medium. However, in light of the results presented in the current study, another possibility is that the increased tolerance to oxidative and osmotic stress observed in these studies were caused by preconditioning through exposure to glycine betaine as a source of xenobiotic stress rather than as a compatible solute. It is well established that yeast cells can be made more stress tolerant through the so-called adaptive response whereby exposure to one type of sub-lethal stress can increase cell tolerance and survival to subsequent exposures of diverse stresses, which would be lethal to non-preconditioned cells (Berry and Gasch 2008; Cañamás et al. 2008; Liu et al. 2011b; Tiligada et al. 1999).

There are additional reports in the literature of both proteinogenic and non-proteinogenic amino acids causing growth inhibition in yeasts. L-lysine has been shown to inhibit growth in *S. cerevisiae* (Sumrada and Cooper 1976, 1978; Thomas and Ingledew 1994; Watson 1983). These reports are noteworthy as *S. cerevisiae* cannot utilise L-lysine as a nitrogen source (Brady 1965), which is analogous to the earlier report by the author that glycine and its *N*-methylated variants often inhibit yeast strains that cannot utilise them as nitrogen sources (Linder 2014). In addition, this author has recently shown that the ω-amino acid β-alanine becomes inhibitory to the yeast *Sch. stipitis* if the gene that encodes the enzyme required for β-alanine assimilation is inactivated (Linder 2019a). In the case of β-alanine, growth of the *Sch. stipitis* mutant was completely inhibited on solid growth media even if a favoured nitrogen source such as L-glutamate was provided. L-lysine inhibition of *S. cerevisiae* has also been shown to depend on the availability and quality of nitrogen sources (Thomas and Ingledew 1994; Sumrada and Cooper 1976). Carbon source availability on the other hand appears to determine the inhibitory potential of L-lysine during *S. cerevisiae* post-exponential growth whereby non-fermentable carbon sources relieve L-lysine-mediated inhibition (Watson 1983). Carbon source-dependent effects on the inhibitory potential of the dipeptide L-carnosine has also been observed in *S. cerevisiae* (Cartwright et al. 2012). The inhibitory effects of glycine and its *N*-methylated variants were not assayed with respect to carbon source in the present study and remain to be established. However, it should be noted that *K. pastoris* was previously shown to be insensitive to L-carnosine (Cartwright et al. 2012).

Lastly, it remains to be determined whether the inhibitory effects by glycine and its *N*-methylated variants described here are of biological relevance and would be observed in nature. The inhibitory effect is thus far only discernable under conditions of nutrient limitation or with certain nitrogen sources. It does raise the possibility of plants using glycine betaine not only as a compatible solute but also as an antifungal compound. None of the budding yeast species assayed in the present study are known to be phytopathogenic but there are related species belonging to the genus *Eremothecium* that are known pathogens of fruit, soy and cotton (Ashby and Nowell 1926). In addition, budding yeasts are closely associated with insects in nature where yeasts serve not only as a critical source of essential nutrients for insects but also in many cases colonise the digestive tracts of insects (Blackwell 2017). Such yeast endosymbionts could potentially aid the insect host in digestion of plant biomass as well as in the detoxification of plant insecticidal defence compounds. This raises the intriguing possibility that plants may produce glycine betaine in part to inhibit yeast growth and thereby defend against predation by herbivorous insects. In conclusion, it is clear that the unexpected growth inhibition by glycine and other amino acids is beginning to reveal a hitherto unexplored dimension of yeast physiology and ecology.
